# Iris: Interactive all‐in‐one graphical validation of 3D protein model iterations

**DOI:** 10.1002/pro.3955

**Published:** 2020-10-19

**Authors:** William Rochira, Jon Agirre

**Affiliations:** ^1^ Department of Chemistry, York Structural Biology Laboratory University of York York UK

**Keywords:** graphics, interactive, model building, plugin, Python, validation

## Abstract

Iris validation is a Python package created to represent comprehensive per‐residue validation metrics for entire protein chains in a compact, readable and interactive view. These metrics can either be calculated by Iris, or by a third‐party program such as *MolProbity*. We show that those parts of a protein model requiring attention may generate ripples across the metrics on the diagram, immediately catching the modeler's attention. Iris can run as a standalone tool, or be plugged into existing structural biology software to display per‐chain model quality at a glance, with a particular emphasis on evaluating incremental changes resulting from the iterative nature of model building and refinement. Finally, the integration of Iris into the *CCP4i2* graphical user interface is provided as a showcase of its pluggable design.

## 1 INTRODUCTION

Macromolecular structure determination primarily involves building an atomic model that best fits experimentally‐observed data from practical methods, such as X‐ray crystallography (MX) and electron cryo‐microscopy (cryo‐EM). At every step of the structure solution pipeline loom unavoidable uncertainties, from the experimental errors introduced in the early stages, to the subjective decisions made during model building. The intertwined steps of refinement and validation at the end of the process play a crucial role in mitigating against this.

Refinement and validation are performed with the help of validation metrics, which provide information about various aspects of the atomic model. They may pertain just to small sections of the model (local criteria) or to the model as a whole (global criteria). The calculation of validation metrics may require only a model, or a model and experimental data (reflection data in the case of MX). Model‐only metrics inform about aspects such as the geometric plausibility of the atomic model as a standalone entity, covering deviations from ideal bond lengths, angles, planes or dihedrals. These geometric analyses result in the detection of outliers: arrangements of atoms that are rare and deemed unlikely to occur, which are either the result of an improbable but true feature of the protein structure, or an error in the protein model. The only way to distinguish between these two possibilities is to compare the atomic model to the experimentally derived electron density, to assess the likelihood that a particular set of atoms is modeled correctly, given the data. Judgments like these are typically made by manually reviewing the questionable area in molecular modeling packages like *Coot* ^1^ or *CCP4MG*,^2^ but can also be helped by local reflections‐based metrics, which take the experimental data into account, such as the Debye–Waller factor (B‐factor) and measures of electron density fit quality. The most commonly used measures of local fit quality are the real space R and real space correlation coefficient, both of which have been demonstrated to show individual biases in assessing the accuracy of a model.^3^

Today, these validation metrics can be produced either by validation‐specific software within software suites like *CCP4* ^4^ and *PHENIX*,^5^ independent web services, or by options and plugins in molecular modeling packages. The number of different routes for model validation has exploded in recent years, having developed from nothing just a few decades ago. Indeed, there is an ever increasing demand for new validation metrics and better refinement procedures,^6^ one most certainly fuelled by periodic realizations that the models in the Protein Data Bank (PDB) are not always perfect.^7^, ^8^, ^9^, ^10^, ^11^, ^12^

In the early days of macromolecular crystallography, refinement was an impossibility; the necessary computational power was simply not available. It was not until 1971 that Robert Diamond published the first automated least‐squares refinement algorithm.^13^ At this stage, the only available validation metrics were global indicators, including resolution and R‐factor.

The introduction of restraints and constraints to the least‐squares refinement process in software—both in small molecule^14^, ^15^ and macromolecular^16^, ^17^ crystallography—brought a significant leap forward by reducing the size of the least‐squares matrix most programs used for their minimization calculations, thus reducing the computational requirement of model refinement. These restraints—ideal bond lengths, angles, planarities and sometimes also torsions angles—did more than just keep the whole process stable; they were about to become very useful metrics to flag up geometric distortions in a protein model. Those distortions may either be a consequence of modeling errors, thus the model should be inspected and corrected, or the product of genuine chemical interactions, meaning the model should be inspected and respected.

As further developments were made, and the amount of computing power available to crystallographers increased exponentially, so too did the amount of available software for macromolecular structure determination. The 1990s saw the inception of the first validation software suite, *PROCHECK*,^18^ which produced a number of summary outputs, including a page containing residue‐by‐residue plots of stereochemical analyses. Though basic, these local analyses proved exceptionally useful to users, providing immediate direction toward areas of the model that were likely to be in need of further refinement or review.

Similarly, the *WHAT IF* ^19^ check report, *WHAT_CHECK*,^11^ performed an array of geometric validation calculations, including some analyses that were not available in *PROCHECK*, for example, unsatisfied donors and acceptors, and suggested side‐chain flips.^20^

In 2004, *Coot* ^1^ took interactive output one step forward from that of *O*,^21^ adding scrollable self‐updating charts as the result of its comprehensive array of integrated validation tools. These included residue‐by‐residue geometric and reflections‐based analyses in the form of pop‐up interfaces. Many of these analyses were based on the *Clipper* C++ libraries.^22^

*MolProbity*,^23^ which produces high‐quality geometric analyses of protein models using their proprietary hydrogen‐placement and all‐atom contact analysis, quickly became and still is one of the most ubiquitous pieces of validation software today. *MolProbity* defines itself as a “structure‐validation web service,” and in addition to the web‐based *MolProbity* servers that produce geometry‐based validation metrics reports, the *MolProbity* libraries are also found in suites like *CCP4* and *PHENIX*. In these implementations, the *MolProbity* server is run locally to calculate the metrics on the back‐end, which are then used by the package‐integrated validation software to generate a report to be shown to the user.

*PHENIX's Polygon* ^24^ provided a way to graphically represent any combination of the available validation metrics meaningfully, in a single view, by plotting multiple quality indicators alongside one another from a shared origin. The one‐shot view of a model's overall quality, combined with the use of percentiles for context, proved very successful and has since inspired other multi‐metric reports (vide infra).

In January 2014, the Worldwide Protein Data Bank partnership (wwPDB) introduced the *OneDep* system,^25^ designed in part to provide “preliminary validation reports for depositor review before deposition.” This incorporated the well‐known summary quality sliders, featured on the summary page for every structure in the PDB, which show a model's percentile rankings for a number of whole‐model validation metrics. The full validation report also contains residue sequence plots which flag geometry outliers.

Each of the pieces of software mentioned so far has brought something new and valuable to the field (Table 1). But, owing to the differences between them, a typical workflow will often involve running different programs—for example, *Coot*, then *MolProbity* or *Polygon*, and finally the *wwPDB* validation server—to obtain the desired array of metrics and paint a complete picture of the outcome of refinement.

**TABLE 1:** An overview of some of the validation tools mentioned

Software	Geometric analysis	Density fit analysis	Per‐residue analysis	Supports integration	All‐in‐one graphics	Interactive
Coot (validation menus)	Yes	Yes	Yes	Yes	No	Yes
MolProbity (web server report)	Yes	No	Yes	Yes	No	No
Polygon (comprehensive validation)	Yes	Yes	No	No	Yes	No
wwPDB (validation sliders)	Yes	Yes	No	No	Yes	No
Iris	Yes	Yes	Yes	Yes	Yes	Yes

*Note*: All the programs mentioned have longer run times than Iris, which are exacerbated in some cases by simple, but mandated, manual input. *Coot* performs all the desired analyses, but provides them in individual horizontally‐scrolled bar charts, rather than an all‐in‐one graphic. Similarly, *MolProbity*, which performs excellent per‐residue geometric (but not reflections‐based) analyses, provides its output as a vertically‐scrolled table. *Polygon* and *wwPDB* both provide an all‐in‐one overview for a model, but not one with residue‐by‐residue analyses.

Movement away from manual model building and refinement, and toward an automated iterative process, has been a long‐time target in the field. Since the early 1990s, software like *O*, and the programs working in conjunction with it, like *OOPS*,^26^ have made it possible to automate a significant amount of the building process, requiring reduced user input. The release of the *ARP/wARP* ^27^ software suite, which aimed to produce essentially complete models from electron density maps alone, paved the way for full automation by coupling the model building and refinement processes together. In recent years, this goal has been almost completely realized by software like *PHENIX's AutoBuild*,^28^ which performs many cycles of refinement and rebuilding to automatically produce a relatively complete model. With fully automated systems like *AutoBuild*, the latest model file can be exported at each refinement iteration, enabling the user to follow the progress of the automated procedure by comparing models from different stages in the overall refinement process. And a useful way of tracking progress is by seeing their validation results side by side.

Novel validation software not only need to be able to calculate both model‐only and reflections‐based analyses at a per‐residue level, but also to be compatible with the recent advances in automation, by having the capacity for integration within new and existing pipelines as an automated task with, ideally, minimal run time.

Iris is a pluggable standalone validation software designed to address the specific needs described here: to provide an all‐in‐one package that calculates its own per‐residue validation metrics—but also allows the incorporation of metrics from other validation services such as *MolProbity*—and displays them in a compact, interactive graphical interface that enables at‐a‐glance comparison between stages of automated model building, and finally, that runs quickly enough to be used either interactively or at the end of a pipeline with imperceptible time penalty.

In the present work, we will discuss the rationale behind the design of Iris's graphics, how its metrics compare to those calculated by other programs such as *MolProbity* and *Coot*, and introduce, as an example, the implementation of our component into the *CCP4i2* ^29^ graphical user interface.

## 2 METHODS

### 2.1 *Component design*

Python was the language of choice for the Iris validation package. Increasingly prevalent in the field, the Python interpreter is a component of all the major crystallographic software packages. Python code is naturally easy to read and write, and because the Iris code was written specifically with maintainability and customizability in mind, it is especially easy for anyone who wants to use the package to edit the source code for their needs. The built‐in metrics calculations are based on the fast *Clipper* ^22^ libraries, thanks to the *Clipper‐Python* C++ bindings.^30^ The Iris module also hooks C++ functions from libraries like *NumPy* ^31^ and the *Computational Crystallography Toolbox* (*CCTBX*),^32^ providing the computational efficiency of strongly typed C++ code, combined with the simplicity of a scripting language like Python. Despite reaching its official end‐of‐life date on January 1, 2020, Python 2 is still the only version available in some crystallographic packages, and such is the case of the *CCP4* suite. Consequently, Iris was written to be compatible with both Python 2 and Python 3.

The Iris validation package has two major components: the metrics module, responsible for the back‐end validation analyses, and the interface module, which generates the front‐end user‐interface.

### 2.2 *Metrics*

The validation metrics chosen for the Iris metrics generation module are those that are most commonly selected in a typical workflow. Based on the class cascade from the *Clipper MiniMol* library (Figure S1), the metrics were implemented with the goal of producing the most accurate results possible with minimal run time. The core analyses can be broken down into three categories: B‐factors, geometry, and electron density fit.

B‐factor analyses are performed by taking the values directly from the *Clipper MiniMol* object. The B‐factor for each atom is listed within the model (coordinates) file, and is loaded as an attribute of each *Clipper MAtom* object upon initialisation. For each residue, the metrics module calculates the minimum, maximum, mean, and *SD* of the B‐factor values of its constituent atoms.

The geometry calculations in Iris include bond length and torsion angles, which are used to analyze backbone conformation (Ramachandran likelihood) and side‐chain conformation (rotamer likelihood). The bonds geometries themselves are calculated with simple matrix calculations using atom coordinate data from the *MAtom* objects. To produce meaningful validation metrics, these bond angles are turned into both a continuous probability score and a discrete classification (favored, allowed, or outlier), using reference data. The Richardson lab has published a public repository of reference data for different types of residue geometry, based on thousands of high‐resolution, quality‐filtered protein chains, called *Top8000*.^33^

In the case of backbone conformation, the *Clipper Ramachandran* class already implements the relevant data from the *Top8000* database, accessible through a selection of calculators which output a probability value for a pair of backbone torsion (phi, psi) angles, sampled from the relevant Ramachandran distribution. This value is used as Iris' continuous score metric, and is also directly used to produce the discrete classification, by applying the same thresholds as those used in *Coot*.

In the case of side‐chain conformation, there is no *Clipper* class to do the work. To generate a validation metric from the side‐chain torsion (chi) angles, the data had to be implemented manually. The Richardson lab's rotamer data is provided for each of the rotameric canonical amino acids in two forms: (a) a multidimensional contour grid that maps out the feasible chi space in discrete intervals, plotting a “rotamericity” value at each point; and (b) a set of “central values,” which lists the mean and *SD* of the bond torsions for each recognized rotamer.^34^

The most accurate way to produce a continuous rotamer score would be to implement the raw data from the contour grids with an interpolating lookup function, but this poses two difficulties. The first is that even if the data from these grids are stored in a data structure optimized for multidimensional search like a *k*‐d tree, looking up and interpolating these data for each angle in every residue in a model would elicit an unacceptably long run time. This could be mitigated against by performing the interpolation in pre‐processing, or by omitting it entirely, given that the contour grids are already fairly high resolution. But, the second and more significant difficulty is that these contour grid files are large, totaling 39 megabytes in all. As a consequence, directly loading these data results in a long initial load time and significantly increases the file size of the package; these factors would only be exacerbated if interpolation were implemented in the lookup function. Because of this, the Iris rotamer score is based on the central values data, which have a much smaller footprint. The score is calculated by modeling each of a rotamer's chi dimensions as Gaussian distributions, calculating a z‐score in each dimension, and taking the quadratic mean (Equation 1).

(1) $$\text{Score}=\min_{i}\sqrt{\frac{1}{N}\sum_{n=1}^{N}{\left(\frac{\chi_{n}-\mu_{\chi_{i_{n}}}}{\sigma_{\chi_{i_{n}}}}\right)}^{2}}$$

Equation (1): formula used to calculate a continuous rotamer score from the central values lists. Where *i* is the index that enumerates the recognized rotamers for a residue, *N* is the number of chi dimensions applicable to a particular residue, *χ*_*n*_ is the nth chi angle of the residue, and $\mu_{\chi_{i_{n}}},\sigma_{\chi_{i_{n}}}$ are the mean and *SD* of the indexed rotamer, respectively.

**FIGURE 1:**
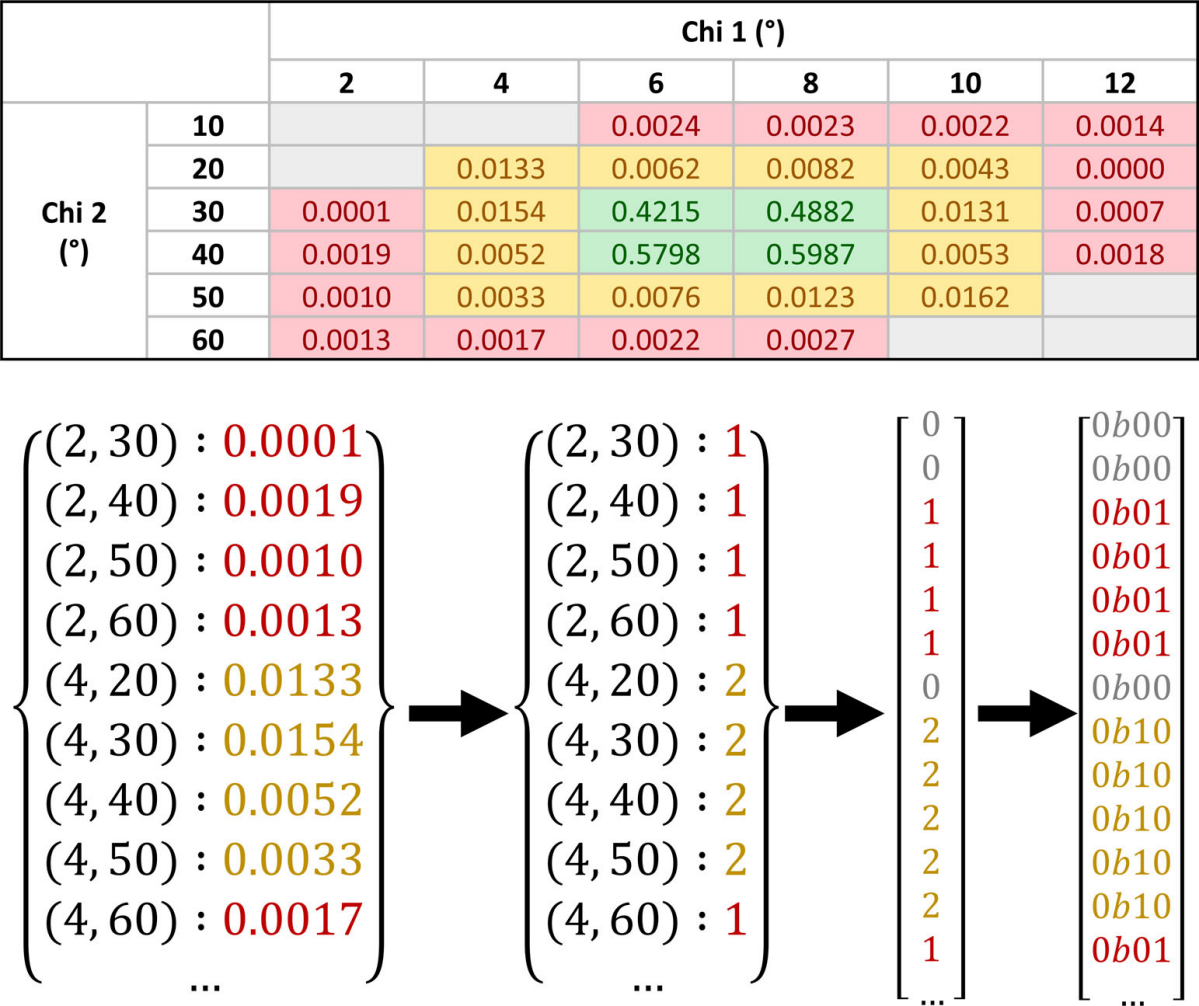
Visualization of the rotamer library compression. The topmost figure shows a contour grid for a hypothetical amino acid with two side‐chain torsion angles. Grid points are colored red for “outlier” values, yellow for “allowed” values, green for “favored” values, and gray for “unknown”—where a coordinate is not listed in the original contour grid file. The bottom figure illustrates the compression process: starting with the conversion from floating point to integer data points, followed by the type conversion from dictionary to integer array, which includes the addition of zeros to represent null data points, and lastly the compression of Python integers to two‐bit binary values. It should be noted that the original contour grid values are given to a much higher precision than is shown here

The rotamer classification, in contrast to the Ramachandran one, is not just calculated by placing thresholds on the continuous score. Instead, a more accurate solution was devised, which involves using a compressed version of the contour grids to achieve a compromise between accuracy and load time (Figure 1). To compress the data, the point values were first converted from the very high‐precision floating‐point values in the contour grids to a low bit‐width integer classification. In order to maintain concordance with the *MolProbity* rotamer analyses, based on the same data, the same thresholds for categorization were applied, that is, let ≤0.3% define “outlier” rotamers, let ≥2.0% define “favored” rotamers, and let values in between define “allowed” rotamers. This reduced the size of the data substantially, but still the most significant factor in the size of the data persisted, which was storing all the coordinates as keys. To maximize the compression, the data for each amino acid were flattened to a unidimensional array of values. This way, the index of each value corresponds to the calculable index of its coordinate in a theoretical ordered array of coordinates (Equation 2). It is only possible to store the data in this way when there is a uniform distance between each point in every dimension, and when there is a value for every possible point in the dimension. In the original data, the latter criterion is not met. To enable flattening, an extra value had to be allocated for “unknown” data points, thus filling every point on the contour grid, and bringing the number of discrete classifications up to four. If these four classifications are treated as integers in the interval [0, 3] each coordinate point only requires a precision of 2‐bits, which means four values can be stored per byte of data. Reducing each classification in the flattened arrays to a 2‐bit value and compressing the result with gzip leads to a file size of 147 kilobytes for the entire library, a 265x reduction from the original data. Upon initialisation of the module, the library loading function can decompress this file and load the library to memory on the millisecond scale, with similarly fast point recall. The compression process is illustrated in Figure 1.

(2) $$\text{index}=\sum_{n=1}^{N}\left[\text{nint}\left(\frac{\chi_{n}-{Χ}_{n_{0}}}{{Χ}_{n_{1}}-{Χ}_{n_{0}}}\right)∙\prod_{m=n+1}^{N}\dim\left({Χ}_{m}\right)\right]$$

Equation (2): formula used to calculate the relevant index in the compressed rotamer library for a given array of chi angles. Where *N* is the number of chi dimensions applicable to a particular residue, *χ*_*n*_ is the nth chi angle of the residue, ***Χ***_*n*_ is the regularly spaced array of chi values known in the nth chi dimension for that residue type, thus (***Χ***_*n*1_ − ***Χ***_*n*0_) represents the width of the spacing in that dimension, and dim(***Χ***_*m*_) is the number of known points in the mth dimension for that residue type. nint is the nearest‐integer rounding function.

Electron density fit scores for each residue are calculated by applying methods of the *Clipper* crystallographic map (*Xmap*) class. Firstly, a map is calculated from the list of reflection data using a fast Fourier transform, and is stored in memory. Then, to score the fit of each atom, the map density at its coordinates is used to calculate an atom fit score (Equation 3). A residue's fit score is calculated by taking the average of the fit scores of its constituent atoms. Average density fit quality for the backbone and side‐chain atoms alone are also calculated.

(3) $${\text{Score}}_{\text{atom}}=-\log\left[\text{NormCDF}\left(\frac{\rho_{\text{atom}}-\mu_{\rho_{\mathrm{map}}}}{\sigma_{\rho_{\mathrm{map}}}}\right)\right]$$

Equation (3): formula used to calculate the density fit score for an individual atom. Where NormCDF is the cumulative density function of the standard Gaussian distribution, *ρ*_atom_ is the electron density at the coordinate of a particular atom, normalized by its proton number, and *μ*_map_, *σ*_map_ are the mean and *SD* of the map electron density respectively.

The final step in the construction of the metrics module was the generation of a percentiles library. This enables the final report to be able to provide a sense of scale to each of the metric values, which is necessary if metrics are going to be displayed alongside one another in a meaningful way. Some metrics would otherwise have to be presented in arbitrary, incomparable units. To generate the library, the metrics generation functions were run for every structure in the *PDB‐REDO* database,^35^, ^36^ in which every model is accompanied by its experimental data. To ensure the library was based on models generated using modern standards, data from structures deposited before 2010 were discarded. The resulting data are based on the analysis of over 66,500 structures and more than 47 million individual residues. The structures analyzed and their respective metrics values were divided into 10 non‐uniform resolution bins. For each bin, thresholds were calculated at each integer percentile for all relevant metrics. The percentile calculations were also performed for all the data together, to be applied to models based on data of unknown resolution. The result of these percentile calculations is a set of highly accurate distributions which can be used to normalize the distributions for any of the continuous metrics.

## 3 INTERFACE

### 3.1 *Graphical panel*

The centerpiece of the Iris report is its graphical panel, which comprises a chain‐view display and residue‐view display presented alongside one another. Both of these are scalable vector graphics (SVGs) that can respond dynamically to user interaction, handled by JavaScript (JS) functions. This SVG/JS format was chosen because it is natively compatible with all modern browsers, even the most basic. Report‐specific SVG and JS code is generated programmatically within the interface module.

### 3.2 *Chain‐view display*

**FIGURE 2:**
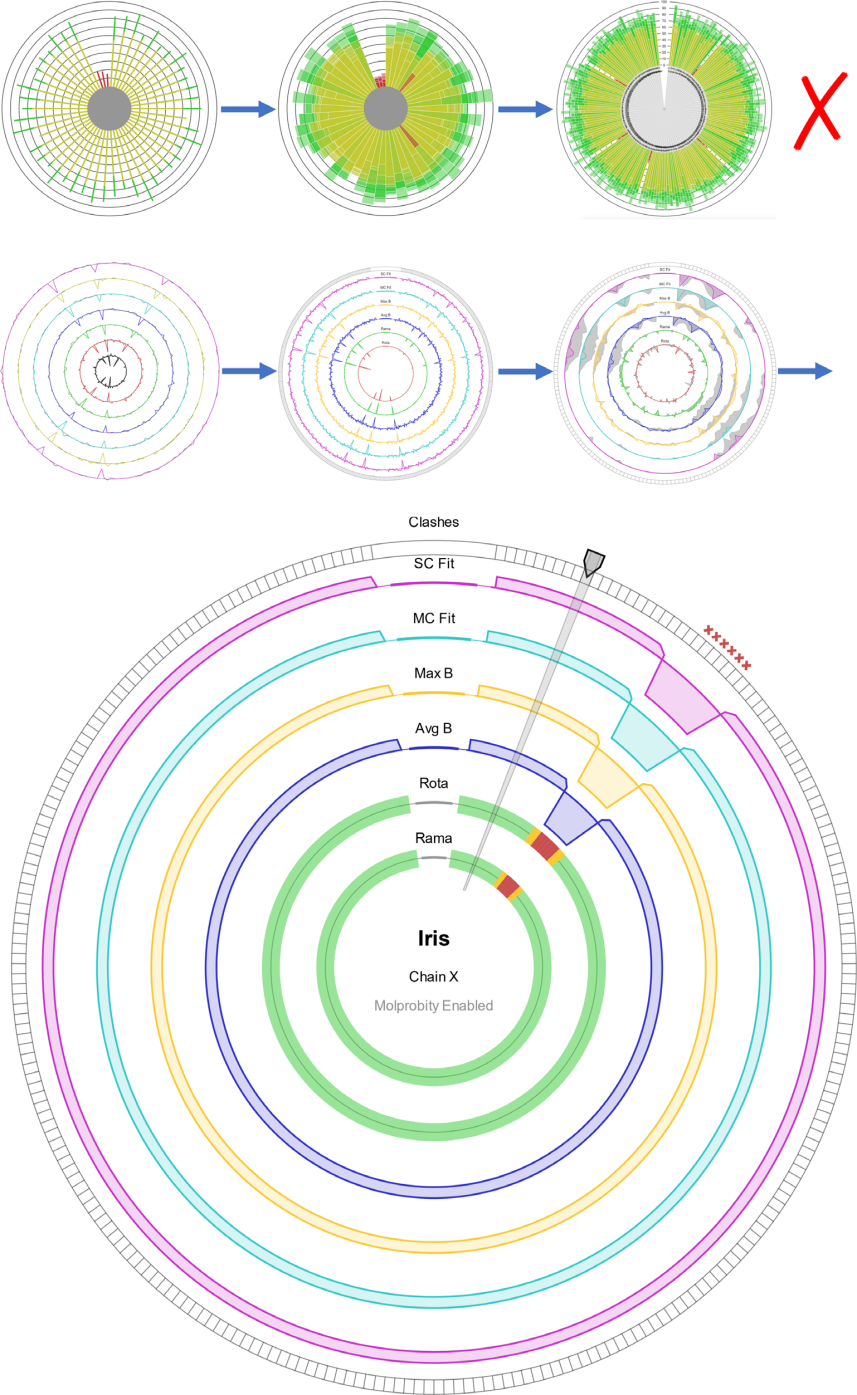
The evolution (top) and final design (bottom) of the Iris chain‐view display. In its first iterations, based on existing residue‐by‐residue displays, the chain‐view was a radial bar chart, with multiple bars stacked on top of one another within a segment. The problem with these initial designs was that in chains with a high number of residues, the chart would become unclear. The third image of the second generation of iterations shows the original “ghosting” implementation. The bottom picture shows an instance of the final design, produced using synthetic data. At the one o'clock position is the residue selector, highlighting an individual residue segment. The patch of 10 residues at the two o'clock position illustrates the indicators of “poor” residues for each feature of the chain‐view graphic. The discrete axes show amber or red segments, the continuous axes show an exaggerated dip toward the center, and *MolProbity* clash indicators appear around the edge

The Iris report was designed around its chain‐view, which illustrates a number of local validation metrics for every amino acid of a protein chain in a single compact display. The graphic went through a number of designs before reaching its final form (Figure 2). In the finalized design, each segment of the circle represents an individual residue, and each of the concentric ring axes represent a different metric. The idea behind this design is that areas of poor protein structure will often cause fluctuations in multiple validation metrics together, making them especially easy to spot. This design is robust to even extremely low or high residue‐counts. Each ring can either represent a continuous metric, with a radial line graph, or a discrete metric, with a traffic light‐based (red, amber, green) segment coloring system. By default, the two innermost rings are discrete representations of Ramachandran and rotamer favorability, and the outer four rings are continuous representations of B‐factors and electron density fit. The axis arrangement can be configured to produce any combination of metrics by editing the definitions file in the root directory of the package.

The continuous axes are individually scaled to collectively emphasize the regions with the poorest validation scores relative either to the rest of the chain or the model, depending on the user's settings. To enable this, the polarity of each axis is set such that the inward direction on the axis represents poorer values for that metric. For example, a higher B‐factor represents a less‐desirable value, so the polarity of the B‐factor axes is inverted, causing areas of higher B‐factor values to appear as troughs, facing toward the center of the plot. Once the polarities have been unified, the axes are skewed to stress the areas on the inward‐facing side of each.

The chain‐view has the ability to show and compare two different versions of the same model, an extremely useful feature in the era of automated iterative refinement pipelines like *AutoBuild*. If Iris is supplied with model data from a prior iteration in addition to the latest, both datasets will be analyzed together, and the collation functions of the report submodule will align the chains and sequences of the two versions using pairwise alignment. This way, the results from both versions can be presented in the same graphic, even if changes have been made to the chains' arrangements or their amino acid sequences. Originally, the two versions were going to be shown concurrently, with the previous dataset represented by a gray shaded “ghosting” area around each axis. Testing showed that this would often make the graphic look too crowded, and some areas would require close examination to understand the changes that had taken place between iterations. In the final design, the different datasets are transitioned between with a toggle switch at the top of the report pane, which triggers an animation that warps between the two model versions, far more intuitively highlighting the areas of greatest change.

The chain‐view is highly extensible, and can be easily adapted to include any metrics added to the metrics module, as well as data from other validation tools. If *MolProbity* analyses are run alongside Iris, clash markers from *MolProbity's* all atom contacts analysis will be displayed around the edge of the circle, and the more accurate *MolProbity* Ramachandran and rotamer outliers will be shown (See *CCP4i2* implementation).

The residue‐selector arm of the chain‐view is used to select any individual residue for more detailed information, to be shown in the residue‐view.

### 3.3 *Residue‐view display*

**FIGURE 3:**
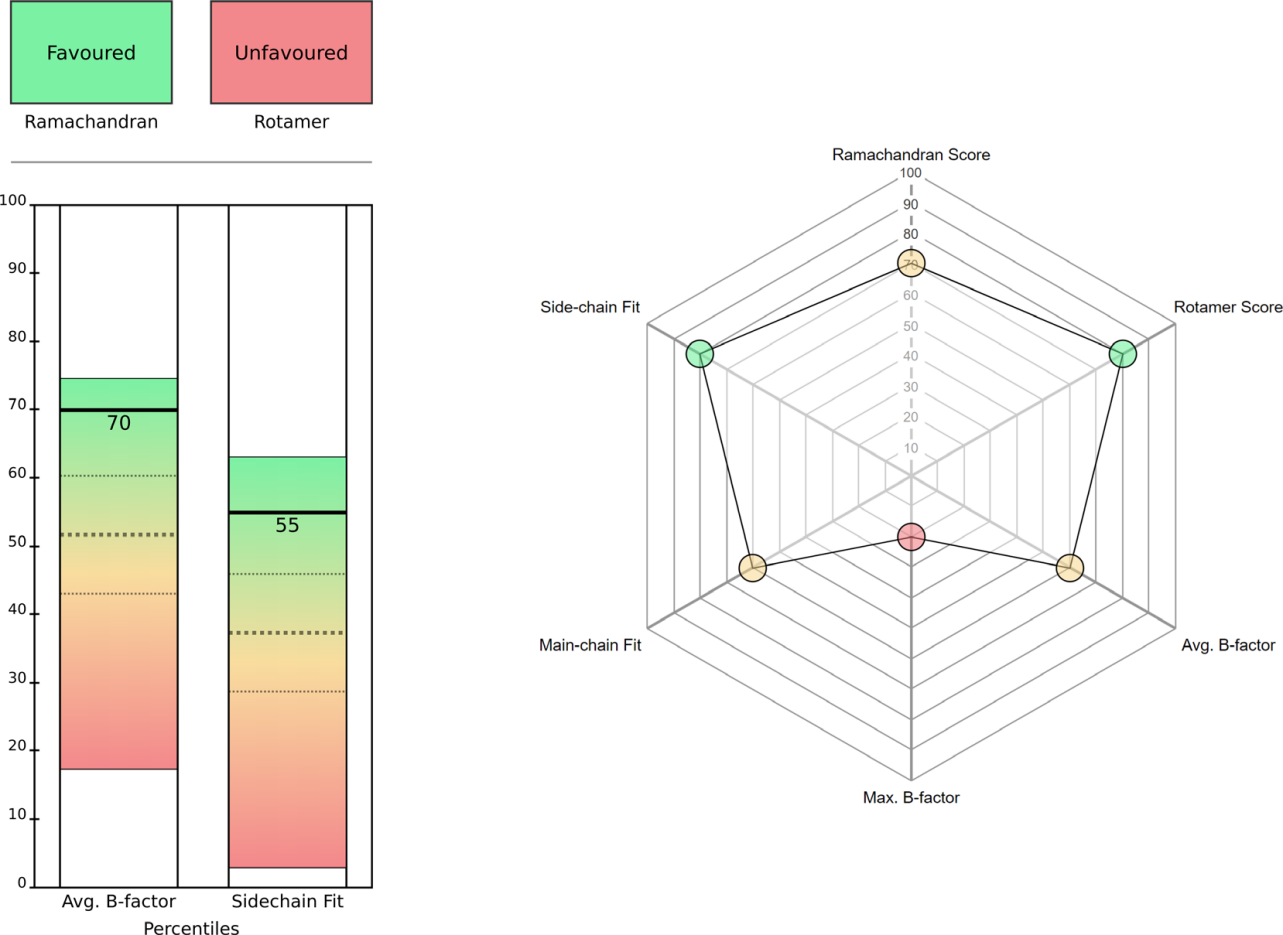
The Iris residue‐view displays: default layout (left) and radar chart (right). In the default layout, the top section contains the discrete metrics, and the bottom section contains the continuous metrics. Of the three dashed lines on each bar, the middle line represents the mean percentile for the selected chain, and the other two lines represent one *SD* from the mean in each direction. The top and bottom of each bar represent the minimum and maximum percentile for the selected chain. The radar chart option shows all the metrics as continuous scores, on the percentile scale. In this chart, the color of the circle corresponding to each metric represents the position of that particular value within its distribution. Hovering over any of these circles produces a pop‐up bubble containing both the absolute and percentile values for a particular metric. The shape of the chart is updated automatically based on the number of metrics selected

The default residue‐view (Figure 3, left) has a grid‐based layout which has one section dedicated to discrete metrics, illustrated with traffic light checkboxes, and another section containing bar charts to represent the percentile values for the continuous metrics, which are the B‐factors and density fit scores. The bar charts also contain individual spectra, which show the minimum, maximum, mean, and *SD* of each of the continuous metrics within the selected chain and model. This way, you get a comprehensive understanding of the quality of a residue, and a chain's distribution of residues, at a glance. The percentile value tells you the quality of the selected residue relative to all other residues of similar‐resolution structures, the position of the marker within each bar's spectrum tells you the metric quality of the selected residue relative to the other residues in the chain, and the distribution of each bar's spectrum tells you the overall quality of the selected chain relative to other similar‐resolution structures.

A radar chart is also available (Figure 3, right), which displays all of the metrics on a continuous percentile scale, including Ramachandran probability and the aforementioned rotamer score.

### 3.4 *Reports*

The Iris validation report is a single HTML file, with the chain‐view and residue‐view implemented as integrated SVG elements. Other linked files include either one or two stylesheet (CSS) files, and the JS files responsible for chart interactivity.

Iris will produce one of two types of report: a full validation report, containing both the graphical panel and further sections of additional validation data, or a minimized report, containing just the graphical panel. The full report provides the ability to test and customize Iris as a standalone entity, or to implement it as an addition to a bespoke Python‐based model pipeline. The intended purpose of the minimized report is to facilitate the integration of Iris within new or existing software suites, to be rendered by the package's own integrated browser, either on its own or via insertion into another HTML page. The simplest way to do this is by using an iframe, to maintain CSS separation.

**FIGURE 4:**
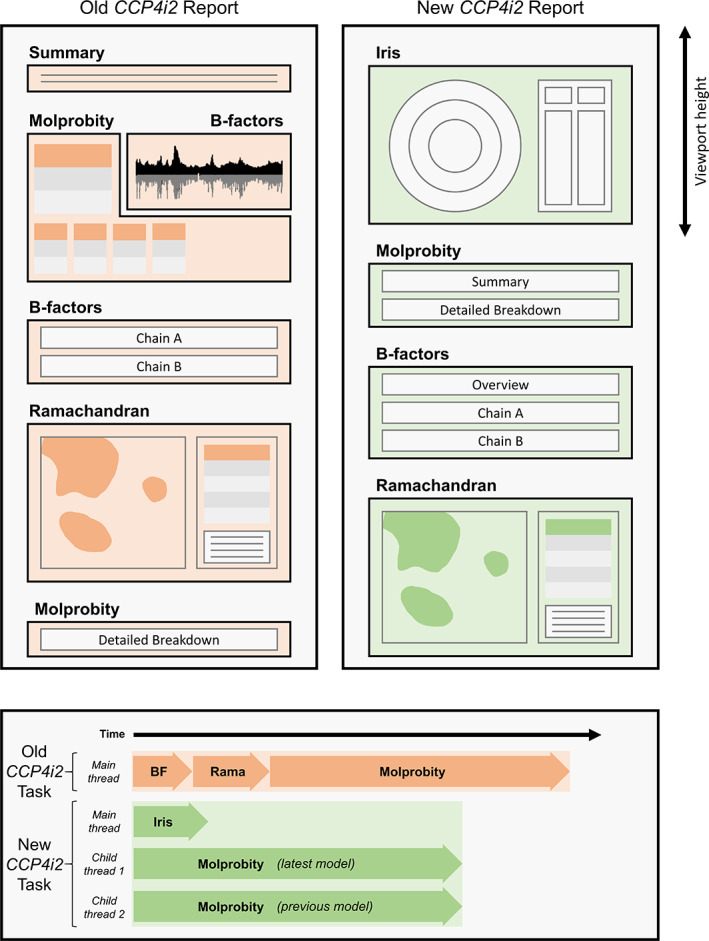
The design (top) and flow (bottom) of the CCP4i2 validation report before and after Iris graphics panel integration with the minimized panel mode. The most noticeable difference to the design is that the Iris graphical panel is now the first view presented to the user when the page is loaded, and fills the viewport to maximize the size of the chain‐view display. The flow of the task has changed more significantly; where the old task performed simple B‐factor and Ramachandran analyses, then executed *MolProbity* analyses and compiled the results all on the same thread, the new version of the task uses Python's multiprocessing library to run concurrent *MolProbity* analyses on separate threads while the Iris calculations are performed on the main thread. This significantly reduces the run time, despite doubling the number of analyses being performed. Because the Iris metrics are all calculated within the same cascade, the task only has to perform one set of (slow) Python loops, as opposed to the serial repeats of loops in the original report, hence the newly‐structured report has a shorter run time both with and without *MolProbity* enabled. Timings are not to exact scale, see results section for accurate timing analysis

*CCP4i2* ^29^ is the latest version of the graphical user interface for the *CCP4* suite, and will be the first package to feature Iris as an integrated validation plugin. The native *CCP4i2* validation routine is the *Multimetric Model Validation* task, which has been renovated with a new structure and design (Figure 4) to feature the Iris graphical panel. Despite the widely‐supported nature of the Iris SVG/JG format, the *CCP4i2* browser originally failed to parse the native Iris JS code, as some of the keywords were unsupported. It was also unable to render the SVG graphics within an iframe. The integrated browsers found in many other crystallographic software packages are similarly outdated. So, a backwards‐compatibility mode was added in which the modern keywords in the Iris JS code are replaced with archaic, but well supported ones, and the CSS is modified such that the Iris HTML can be inserted directly into an existing HTML document without causing any style conflicts. This mode is what is used within the *CCP4i2* implementation of Iris.

At the bottom of the *CCP4i2* validation task is a button that launches the *Coot* software with a “guided tour of issues” raised in the validation report. Because *Coot* and Iris both use the same data and thresholds for the detection of Ramachandran and rotamer outliers, the outliers flagged in the *CCP4i2* validation report directly correspond to those that would be detected in *Coot*, making for a seamless transition from the *CCP4i2* validation report to the *Coot* guided tour.

### 3.5 *Package overview*

The following is an example of the most basic way to generate a standalone Iris report, by importing the generate_report function from the top of the package. The process triggered by calling this function is illustrated in Figure S2.

from iris_validation import generate_report
generate_report (*latest_model_path* = ‘latest.pbd’,
*previous_model_path* = ‘previous.pbd’,
*latest_reflections_path* = ‘latest.mtz’,
*previous_reflections_path* = ‘previous.mtz’,
*output_dir* = ‘Iris_output/’,
*mode* = ‘full’)

## 4 RESULTS AND DISCUSSION

### 4.1 *Metric quality tests*

**FIGURE 5:**
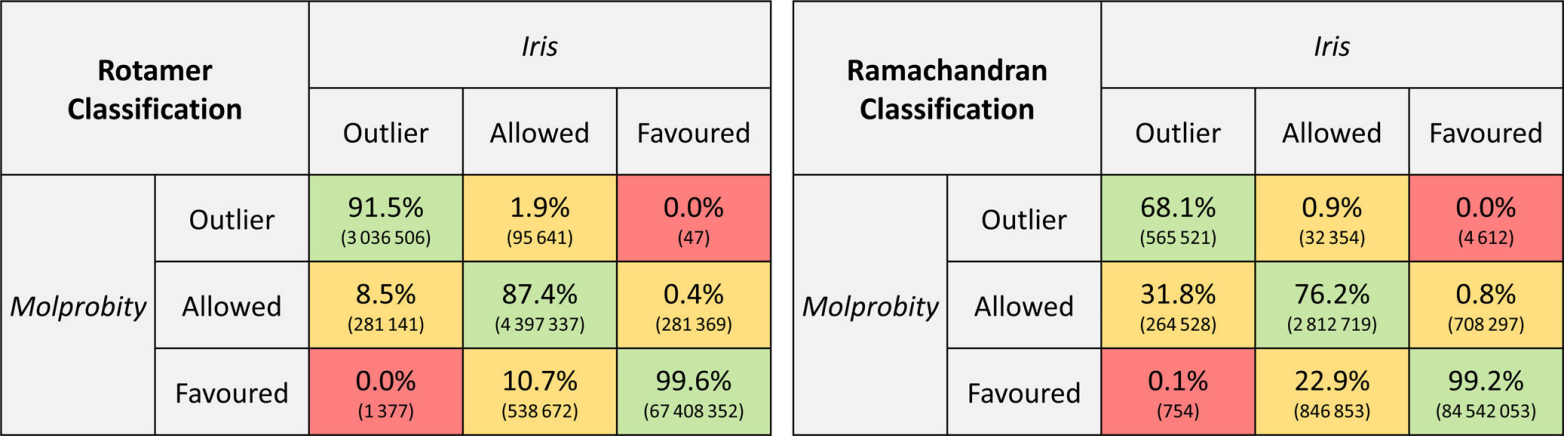
Confusion matrices for Ramachandran (left) and rotamer (right) classification agreements between Iris and MolProbity. Figures in brackets are the number of residues. Percentages are given as a proportion of the sum of each Iris classification. In the case of rotamer classifications, discrepancies between *MolProbity* and Iris arise as a result of the different formats of the reference data; *MolProbity* has access to the entire original dataset, allowing for very accurate interpolation for each case, whereas the compression Iris uses to store the reference data yields slightly less precise classifications, especially at the interfaces between classifications (i.e., borderline cases). Discrepancies in the Ramachandran classifications are partly due to the differing interpolation methods applied by *MolProbity* and Clipper, but more significantly to the fact that the thresholds are arbitrary; and those selected for iris are the ones that are used in Coot, to facilitate the transition between an Iris report and the Coot validation tools. These are not necessarily the same as those used by *MolProbity*

Ramachandran and rotamer classifications were tested against *MolProbity* (Figure 5). Because the thresholds applied for rotamer classification are the same as those applied by *MolProbity*, but those applied for Ramachandran classification were those used by *Coot*, the rotamer classifications show higher agreement with *MolProbity* than the Ramachandran classifications within the outlier and allowed categories. The Iris Ramachandran classifications will be in complete agreement with those from *Coot*.

### 4.2 *User interface tests*

To showcase Iris's functionality, example reports were generated for a number of structures using models from the *PDB‐REDO* database. In these tests, the *PDB‐REDO* refined models were used as the “latest” inputs and the originally deposited models were used as the “previous” inputs.

### 4.3 *Analyzing the structure of a beta‐galactosidase mutant (PDB code 3VD3)*

**FIGURE 6:**
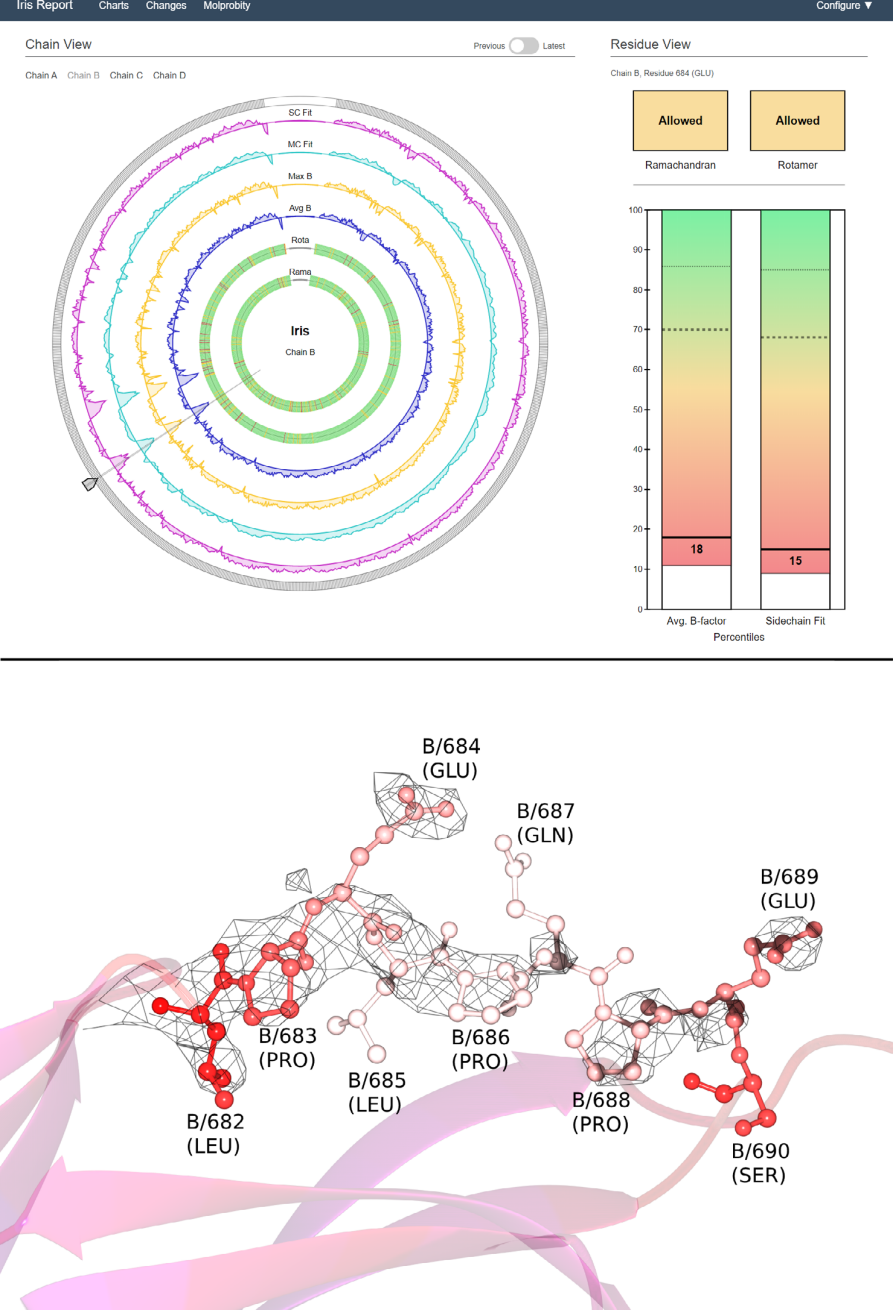
Example Iris report for structure 3VD3 (top) and accompanying model visualization (bottom). The screenshot shows an Iris report for 3VD3, with chain B, residue 684 selected. The version slider in the “previous” position, corresponding in this case to the originally‐deposited model, before refinement by *PDB‐REDO*. The selected chain comprises more than 1,000 residues, demonstrating the robustness of the design to high residue‐counts. For the bottom panel, the model has been colored by B‐factor (blue for low values, red and then white for high relative values) to highlight the mobility of this region. The map shows 2mFo‐DFc density contoured at 1σ; the fact that the map does not cover all the residues at this level hints at the region's mobility and/or disorder

This structure^37^ was chosen due to its high residue count and the fact that the resolution of the experimental data is not high. Looking first at the chain‐view display (Figure 6), the outer axes reveal two troughs around the eight o'clock position in all the continuous metrics, which correspond to the low‐end of the distributions shown on the residue‐view spectra. The first is from B/681–689, in which the selected residue lies, and the second is from B/727–737. These areas both correspond to random coils on the very outside of the molecule, regions which often have quite low fit quality. The two innermost rings reveal some geometry outliers, though not an alarming number given the resolution of 2.80 Å. Their distributions are quite clear: the Ramachandran outliers are mostly spread out, and there is a cluster of rotamer outliers around the nine o'clock position.

Turning to the residue‐view display, the spectra on the bar charts show that this chain has quite high‐quality distributions of the continuous metrics, both with a high mean and low *SD*. However, both distributions have quite low minima, indicating a small number of residues with particularly high B‐factor and poor fit quality. This is likely to correspond to the two troughs seen on the chain‐view display, including the selected residue, which has poor continuous metrics relative to the chain's distribution, and to other models in the percentile bin. Finally, the residue's Ramachandran and rotamer conformations are both in the “allowed” category, indicating unusual conformations for both backbone and side‐chain.

The model visualization shows the random coil corresponding to the first trough on the chain‐view display (B/681–689). Here, the relevant residues are shown in a ball‐and‐stick view with each atom colored by B‐factor. The density has been contoured at 1σ, and clipped around these residues.

### 4.4 *Automated model building: Watching progress and identifying regions for manual intervention*

**FIGURE 7:**
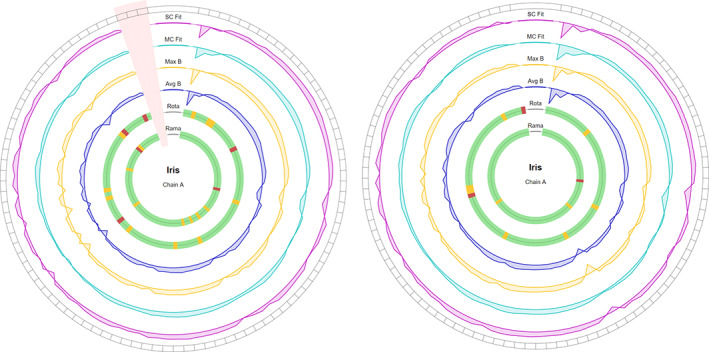
Example chain‐views for the first (left) and last (right) iterations of a model from the Autobuild Protein pipeline (CCP4i2). The pink shaded area on the left Iris plot illustrates an area of missing residues—not modeled by Buccaneer

This case was taken from the “rnase” tutorial that comes bundled with CCP4i2. After phasing the data by molecular replacement with *Phaser* ^38^ using PDB code 3A5E^39^ as model, the *Autobuild Protein* pipeline was launched, which runs alternate iterations of Buccaneer^40^ and *REFMAC5*,^41^ and extracted the coordinates resulting from the first and last iterations of the pipeline. The results obtained by Iris on these two structures can be seen in Figure 7.

The sequence of the first iteration of the model is three residues shorter than in the final iteration, due to Buccaneer being unable to model this section. Pairwise alignment enables Iris to determine that the missing residues are the final three of the chain, which is represented on the chain‐view with the black spots in the segments around the edge of the ring. Looking first at the inner two rings, it is evident that the Ramachandran and rotamer torsion angles improved significantly, with non‐favored Ramachandran residues decreasing from nine to three, and non‐favored rotamers decreasing from fourteen to nine. On the outer four rings, changes are more subtle, and much easier to make out with the live animation (please refer to our Supporting Information Video S1). At around the seven and eight o'clock positions, there is some improvement in both B‐factor and density fit quality. Because Iris emphasizes the poorer areas on the chain, this change appears very slight. More noticeable are the areas of poor quality that have developed between the two versions, for example, a trough developed in all four rings at the third residue, where apparently fit quality and B‐factor were sacrificed in order to swap the rotamer for a more favorable conformation.

### 4.5 *Timings*

**FIGURE 8:**
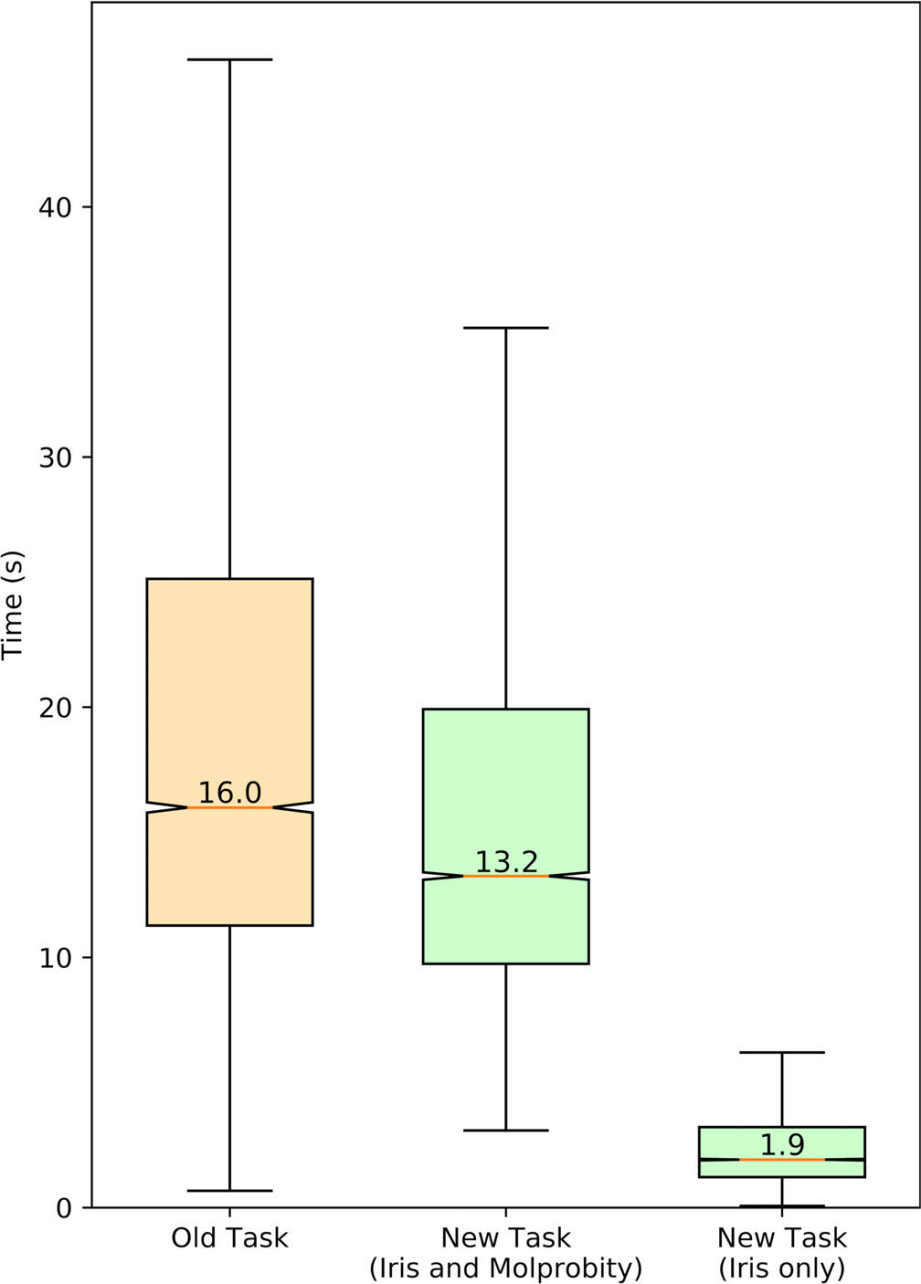
Boxplots illustrating the distribution of average (*n* = 5 repeats) times taken to run models (both coordinates and reflection data) through different versions of the CCP4i2 Multimetric Validation task. The median value of each distribution is labeled

A random selection of 20,000 models from *PDB‐REDO* was run through three different versions of the *CCP4i2* validation task: (a) the old version of the task, with *MolProbity* analyses enabled; (b) the new Iris‐implemented version of the task, with *MolProbity* analyses enabled; (c) the new Iris‐implemented version of the task, with *MolProbity* analyses disabled. It is important to note that the old version of the task only analyses the originally deposited model, whereas the Iris‐implemented task analyses both the original and optimized versions together. The results are shown in Figure 8.

Timings were calculated on an Intel i9‐9900k (eight cores, sixteen threads) at stock frequency, with eight *CCP4i2* instances running in parallel. Because each instance of *CCP4i2* can have up to three intensive processes running at once (one main thread plus two *MolProbity* threads) the processor thread count will have led to bottlenecking at times.

The implementation of the Python 2 multiprocessing module in the *CCP4i2* task is not supported under Windows. Hence, when the *CCP4i2* validation task is run under Windows, *MolProbity* analyses have to be run sequentially on the main thread, without parallelization, the same as in the old task. Because of this, if two models are provided, and *MolProbity* is enabled, validation may take significantly longer than it otherwise would on a unix‐based operating system like Linux or MacOS. Forthcoming updates to the *CCP4* package instating Python 3 will solve this issue in the near future.

## 5 CONCLUSIONS AND FUTURE WORK

Our main aim at this stage was to demonstrate the benefits of using an interactive multi‐metric per‐residue display; the fact that problematic regions in a model create ripples across the different metrics helps spot those parts of a model requiring further attention.

In the near future, *MolProbity* analyses will be implemented directly within the Iris metrics module using the *CCTBX* Python package. This way, the user will be able to choose either Iris or *MolProbity* analyses when using Iris in any context, including as a standalone solution, rather than having to choose an implementation of Iris that makes *MolProbity* analyses available, such as *CCP4i2*.

The Iris metrics module cascade is an ideal candidate for multithreading. Residue analyses could be parallelized for a significant reduction in run time. Unfortunately, the way that Python 2 requires multiprocessing processes to return a class that can be serialized with Python's built‐in serialization module makes this infeasible at the moment. In the longer term, the Iris code will be restructured to realize this goal.

The calculation used for electron density fit score is quite oversimplified for the sake of reducing computational intensity. If other optimizations are made, like multithreading, then more processor time can be spent on more comprehensive density fit calculations. Alternatively, we could incorporate the ability to parse output from programs like *EDSTATS*,^3^ which is bundled with the *CCP4* suite.

Owing to its modularity and portability, we expect to make Iris available to a number of structural biology programs, including *CCP4mg*,^2^ *Coot* ^1^ and *ChimeraX*.^42^ These graphical programs will also provide a 3D view that can be centered upon clicking on individual residues in the Iris report. The mechanism we envision for this task has already been tested in the implementation of *Glycoblocks*,^43^ which communicated the *Privateer* ^44^ carbohydrate validation software and *CCP4mg* through hyperlinks on SVGs.

The most pressing development however is to expand the number of metrics available that can be generated by the Iris metric module. Like with the compressed Iris rotamer library, a compressed library for *CaBLAM* ^45^, ^46^ could be generated and integrated using the Richardsons' group data.^33^ *CaBLAM* C‐alpha evaluation can be useful in the refinement and validation of models derived from cryo‐EM data, which are becoming increasingly prevalent. Support for such data will be added soon, through application of the *Clipper NXmap* class. Other traditional metrics to be included will cover planarity and chirality favorability.

However, all the aforementioned metrics are easily targeted by restrained refinement, potentially devaluing them as validation criteria. A longer, more challenging project will involve the inception of a new set of validation metrics that remain as separate from the refinement process as possible, opening the door to a truly independent evaluation of the quality of a protein model.

## 6 REPRODUCIBILITY AND AVAILABILITY

Iris validation is available from GitHub (https://github.com/wrochira/iris-validation), and soon, as a regular Python package installable with the pip install iris‐validation command. A forthcoming *CCP4* update will distribute the component and its *CCP4i2* ^29^ interface.

The scripts used to generate and test any of the data implemented in the package can be found in the Iris tools companion module within the same repository. Therefore, if any customizations are made to the metrics module, the percentiles library can be regenerated with ease.

## AUTHOR CONTRIBUTIONS

**Will Rochira:** Conceptualization; data curation; funding acquisition; investigation; methodology; software; visualization; writing‐original draft; writing‐review and editing. **Jon Agirre:** Conceptualization; methodology; supervision; validation; writing‐original draft; writing‐review and editing.

## Supporting information


**Figure S1** *Visualization of the metrics module cascade*. Upon initialisation of the *Clipper MiniMol* object from a coordinates file, the *MetricsModel* class is instantiated with a *MiniMol* model object, and iterates through its child chains (*MPolymers*), instantiating a *MetricsChain* object for each. Each *MetricsChain* object iterates through the residues (*MMonomers*) in the polymer, instantiating a *MetricsResidue* object for each, with context (references to their neighboring *MMonomers*) to enable each *MetricsResidue* object to independently perform backbone geometry calculations. Attributes of the *MMonomer* are analyzed, including constituent atoms and bond geometries, to determine whether it represents an amino acid residue; if it does, the rest of the analyses are performed.

**Figure S2** *an overview of the structure of the Iris validation package*. The *generate_report* function at the top of the package first calls the *generate_metrics_model* function from the metrics submodule. This function instantiates a *ReflectionsHandler* object from the reflections submodule, to calculate a map from the reflections file, and then initialises the metrics calculation cascade using the coordinates file. Each *MetricsResidue* object then performs analyses on itself using functions from the utils module, rotamer submodule, and *ReflectionsHandler* object, then runs the calculated metrics through the percentiles submodule. Once the metrics calculations are finished, the *generate_report* function calls the *build_report* function of the interface module, which generates the graphics and produces the finished report.

**Video S1** Iris validation
