# Supplementary figures and images for: Iris: Interactive all‐in‐one graphical validation of 3D protein model iterations
Source: Protein Sci. 2020 Oct 19;30(1):93–107. doi: 10.1002/pro.3955 (PMC7737763; doi:10.1002/pro.3955)

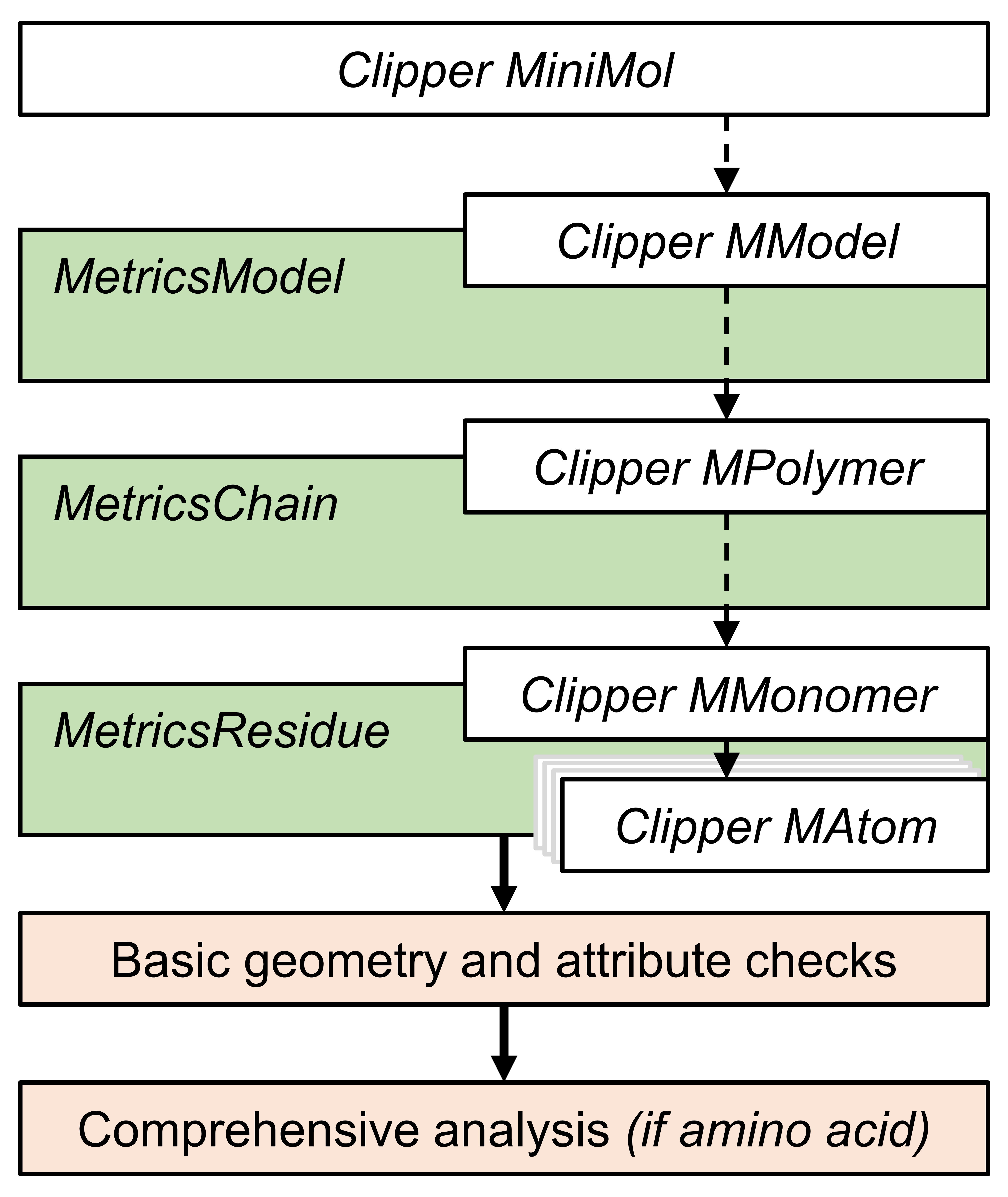

Supplement: Supplementary file 1 — Figure S1 Visualization of the metrics module cascade. Upon initialisation of the Clipper MiniMol object from a coordinates file, the MetricsModel class is instantiated with a MiniMol model object, and iterates through its child chains (MPolymers), instantiating a MetricsChain object for each. Each MetricsChain object iterates through the residues (MMonomers) in the polymer, instantiating a MetricsResidue object for each, with context (references to their neighboring MMonomers) to enable each MetricsResidue object to independently perform backbone geometry calculations. Attributes of the MMonomer are analyzed, including constituent atoms and bond geometries, to determine whether it represents an amino acid residue; if it does, the rest of the analyses are performed. [file PRO-30-93-s001.tif]
